# Auto-abdominoplasty: A unique case of self-surgery and its implications

**DOI:** 10.1016/j.ijscr.2025.111932

**Published:** 2025-09-11

**Authors:** Hazem M. Ali, Ahmad N. Mohammad, Mohammed H. Mohammed, Radwan Al-Kattan, Tarek Houssien, Doha Hajjah

**Affiliations:** a Al-Madina Hospital, Damascus, Syria; b Damascus University, Damascus, Syria

**Keywords:** Case report, Abdominoplasty, Self-surgery

## Abstract

**Introduction and importance:**

Self-surgery is an extreme deviation from standard medical practice. The motivations, technical challenges, and psychological implications of such an act present a unique medical and ethical dilemma.

**Presentation of case:**

We present the case of a 40-year-old plastic surgeon who suffered from abdominal sagging and previous heart problems. Due to his previous medical history, several of his colleagues refused to perform an abdominoplasty on him, despite the anesthesiologists agreeing to safety perform the surgery under epidural anesthesia, which prompted him to perform the surgery himself.

**Clinical discussion:**

This surgery differs from other self-surgeries reported in the medical literature in terms of the purposes of which it was performed. It represented the surgeon's desire to perform this cosmetic surgery safely on a specific category of patients.

**Conclusion:**

Patients with a significant medical history shouldn't be deprived of the opportunity to undergo cosmetic surgery, as this can be performed in accordance with an anesthetic consultation.

## Highlights

• First case of self-performed abdominoplasty under epidural anesthesia.
• The procedure reflecting a unique case but raising concerns about self-care boundaries.
• Safe epidural use for patients with significant cardiac history.

## 1 Introduction

Self-surgery, also known as auto-surgery, refers to the highly unusual and often perilous act of an individual performing a surgical procedure on their own body [1]. While generally an extremely rare occurrence in modern healthcare, it has been documented throughout history, emerging primarily from dire circumstances where professional medical assistance is completely out of reach. These procedures are typically undertaken as a last resort, driven by a life-threatening medical emergency [2], a profound lack of medical infrastructure [3,4], or, in some rare cases, as a radical form of self-experimentation by medical professionals [5,6].

Abdominoplasty, commonly known as a tummy tuck, is a sophisticated surgical procedure aimed at reshaping and firming the abdominal area. This procedure involves the removal of excess skin and fat, often a result of significant weight loss, pregnancy, or the natural aging process. Simultaneously, the underlying abdominal muscles, which may have become weakened or separated (diastasis recti), are tightened to create a flatter, more contoured midsection [7].

Traditionally, tummy tucks have been performed under general anesthesia, where the patient is completely unconscious throughout the surgery. While effective, general anesthesia carries certain inherent risks and can lead to side effects such as nausea, prolonged recovery, and a higher incidence of blood clot formation [8]. This has led to the exploration and increasing adoption of alternative anesthetic techniques, with epidural anesthesia emerging as a compelling option [9].

This case is described in accordance with criteria of SCARE [10].

## 2 Presentation of case

### 2.1 Patient information

A 40-year-old male with history of morbid obesity (BMI 45.8), hypertension, diabetes, and heavy smoking suffered an acute inferior MI four years ago, treated with PCI (RCA and circumflex stenting). Post-MI, he achieved significant weight loss (145 kg → 90 kg, BMI 28.4) with improved BP and HbA1c control. The resultant abdominal skin laxity caused physical and psychological distress, leading him to seek abdominoplasty. Multiple plastic surgeons declined due to cardiac risks, prompting him to perform the procedure himself. After multidisciplinary evaluation, surgery was conducted under thoracic epidural anesthesia (ropivacaine) for its favorable cardiac profile.

### 2.2 Surgery

The patient was admitted to the hospital the day before surgery. The entire area from the neck to the knees was sterilized with povidone the night before surgery and the morning of surgery, while wearing a sterile coat. The patient (the same doctor) performed the traditional preoperative wash, and the entire surgical area was then sterilized again. Prior to induction of anesthesia, 500 ml of Ringer's lactate solution and 8 mg of ondansetron were administered intravenously. Pre-anesthesia blood pressure was 140/85 mmHg, and pulse was 74/min. Based on the decision of the hospital administration there was a plastic surgeon among the surgical staff, qualified to intervene in the event of any complications during the surgery.

**Fig. 1:**
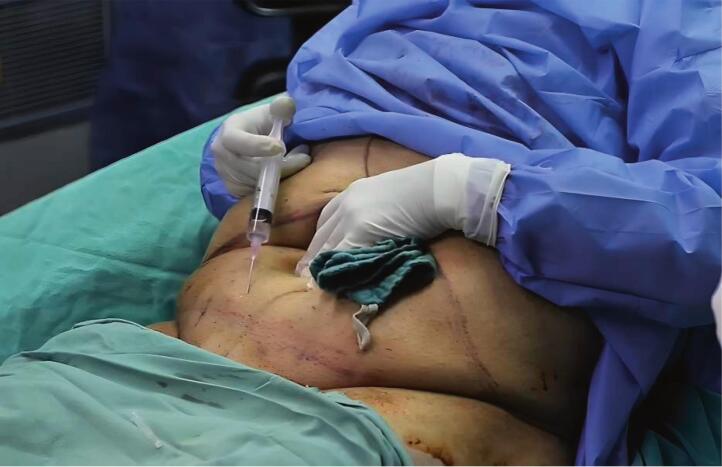
Subcutaneous filtration for surgical incision sites.

**Fig. 2:**
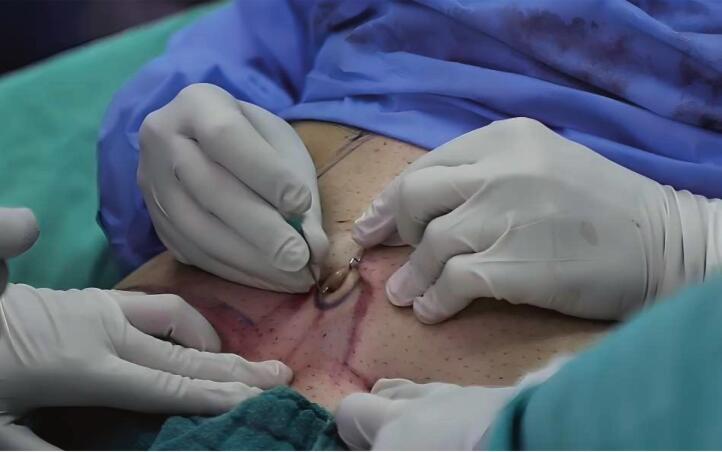
Umbilicus suspension with two hooks.

**Fig. 3:**
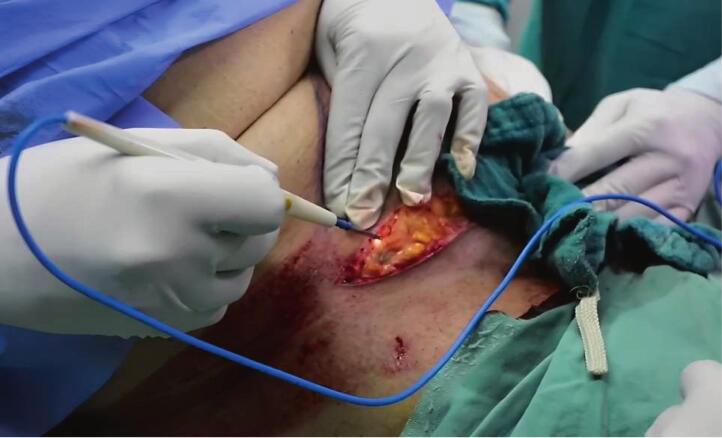
Beginning of dissection using an electric coagulator.

**Fig. 4:**
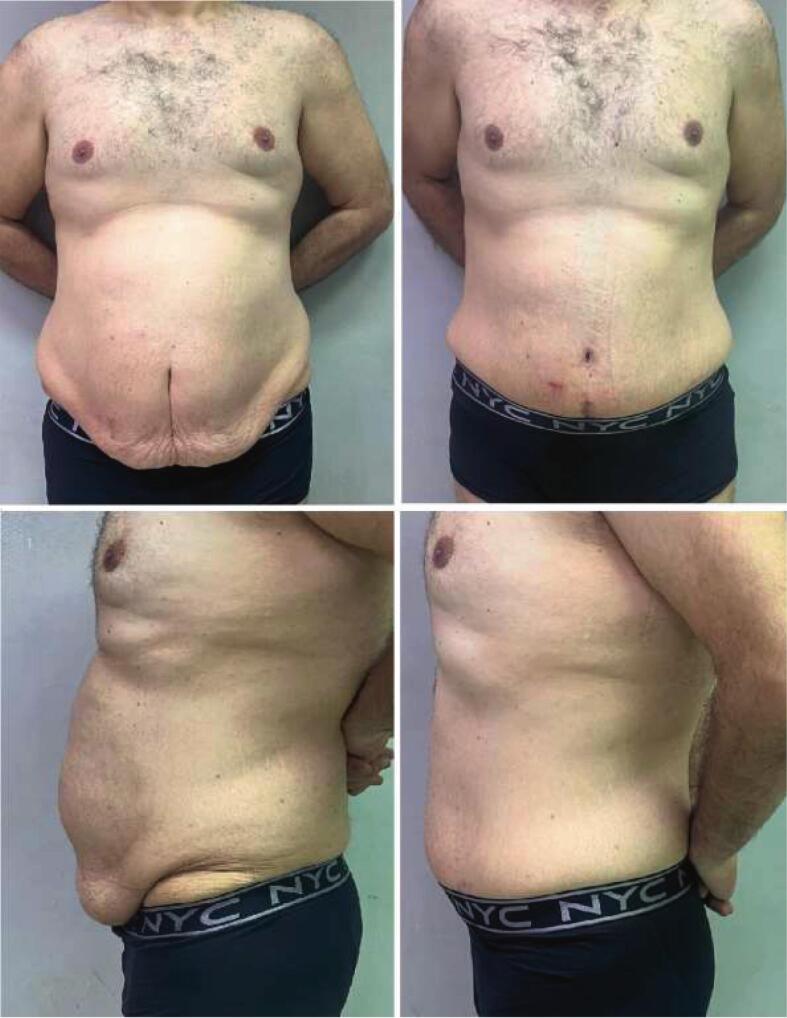
Pictures showing the difference before (left) and after surgery (right).

After epidural anesthesia, subcutaneous filtration was performed using 50 ml of 2 % lidocaine with 1 ml of adrenaline diluted in 0.9 % saline (Fig. 1). Two hooks were used to suspend the umbilicus (Fig. 2), and an 11-scalpel was used to isolate the umbilicus after elevating it with hooks. An incision was made on the inferior abdominal crease between the anterior superior iliac spines, in addition to an incision on the superior portion of the sagging flap. The dissection began from the right toward the center and then from the center toward the left. The dissection was performed above the level of the muscular peritoneum using an electric-coagulator (Fig. 3). The supra-umbilical area was then dissected to the xiphoid bone, between the level of the muscular aponeurosis and the subcutaneous fat. The new umbilicus was determined by approximating the superior edge of the excision to the inferior edge. The umbilicus was sutured in its new location using a 4/0 monocryl cutting suture. The subcutaneous layer was sutured using a 0 round Vicryl suture. The skin was closed using a metal stapler. He was discharged from the hospital 6 h after surgery and returned to his medical practice 5 days later. The metal staples were removed after 10 days with a re-evaluation of the success of the procedure (Fig. 4).

20 min post-anesthesia induction, BP dropped to 85/46 mmHg with HR 48/min, treated with IV atropine 0.5 mg, ephedrine 6 mg, and Ringer's lactate. After 5 min supine, surgery resumed. At 45 min, mild dizziness occurred (BP 100/65 mmHg), managed with 5 min recumbency.

## 3 Discussion

Historically, the motivations behind self-surgery have been diverse, ranging from pragmatic necessity to scientific curiosity. The annals of medical history are punctuated by extraordinary tales of self-surgery, each highlighting unique circumstances and motivations. These cases, though few, provide invaluable insights into human courage and resourcefulness.

Joannes Lethaeus, a 17th-century Dutch blacksmith, performed self-lithotomy for bladder stones, documented by Dr. Nicolaes Tulp [3]. Dr. Evan O'Neill Kane pioneered self-surgeries (1919–1932) including finger amputation, self-appendectomy, and hernia repair to demonstrate local anesthesia's efficacy [5,6]. Dr. Leonid Rogozov famously performed an auto-appendectomy in Antarctica (1961) during a blizzard when evacuation was impossible [2]. Similarly, Dr. Jerri Nielsen self-biopsied her breast cancer while stationed in Antarctica [4]. Modern literature further contextualizes this case. Self-experimentation persist in medicine, often driven by necessity or innovation, but raises concerns about objectivity and safety. Physicians undergoing self-treatment may underestimate risks due to psychological dual-role conflict [11].

Unlike previous reports, this case was conducted in a well-equipped specialized hospital with a skilled team. The surgeon aimed to show that patients with cardiovascular, respiratory, or neurological risks—often denied cosmetic surgeries like abdominoplasty despite psychological need—can safely undergo these procedures with proper preventive measures and tailored anesthesia (e.g., epidural), minimizing complications. Additionally, the surgeon sought to personally experience epidural anesthesia and recovery to better understand patient perspectives.

The two episodes of hypotension during surgery were expected and managed smoothly and did not interfere with the surgical procedure. Epidural anesthesia itself blocks sympathetic nerve fibers, causing hypotension and bradycardia. The patient's position (sitting) during surgery also impeded adequate venous return to the heart and brain. Furthermore, the patient was performing the surgery, which required focus and energy.

This case underscores profound ethical tensions between patient autonomy and professional boundaries. While the surgeon's self-operation arose from legitimate clinical and psychological distress, it contravened global medical standards—including the WMA Declaration of Geneva and AMA Code of Ethics—which prohibit self-treatment outside emergencies due to risks of impaired judgment and inadequate oversight. The technical success of the procedure does not justify the method; rather, it sets a perilous precedent that could encourage unsafe practices. Crucially, this case should not be interpreted as endorsing self-surgery, but it does challenge systemic biases in patient eligibility by demonstrating that high-risk individuals *may* tolerate abdominoplasty under optimized anesthesia. However, such interventions must never bypass multidisciplinary evaluation, as anesthetic clearance alone cannot guarantee surgical safety in comorbid patients. Ultimately, this report should catalyze evidence-based guidelines for equitable—yet rigorously vetted—access to cosmetic procedures, rather than legitimizing self-operation.

## 4 Conclusion

This case demonstrates the technical feasibility of epidural abdominoplasty in high-risk patients but must not be misconstrued as endorsing self-surgery. The ethical breaches and potential harms outweigh the individual benefits. Future efforts should focus on: developing evidence-based guidelines for cosmetic surgery in comorbid patients, integrating anesthesia, surgery, and psychology expertise. Strengthening institutional safeguards to prevent self-treatment outside emergencies. Finally, promoting equitable – but not unconditional – access to elective procedures, with rigorous risk-benefit analysis.

## Abbreviations

BMI: body mass index
MI: myocardial infraction
PCI: percutaneous coronary intervention
BP: blood pressure
HR: heart rate

## Informed consent

The patient, a board-certified plastic surgeon, provided explicit written consent for the publications of this case report, including details of the surgical technique, perioperative management, and the clinical outcomes. He understands that this report may generate medical, ethical discussions regarding self-operated procedures.

## Consent

The patient, a board-certified plastic surgeon, provided explicit written consent for the publications of this case report, including details of the surgical technique, perioperative management, and the clinical outcomes. He understands that this report may generate medical, ethical discussions regarding self-operated procedures. A copy of the written consent is available for review by the Editor-in-Chief of this journal on request.

## Ethical approval

Not required for case reports. Single case reports are exempt from ethical approval.

## Guarantor

Mohammed H. Mohammed.

## Registration of research studies

Not applicable in our case.

## Sources of funding

This research did not receive any specific grant from funding agencies in public, commercial, or not-for-profit sectors.

## Author contribution

MHM, ANM, TH, DH: who wrote, original drafted, edited, visualized, validated, literature reviewed the manuscript.

HMA: Plastic surgeon who performed surgery.

RA: Anesthesiologist in this surgery.

## Declaration of competing interest

No conflicts of interest are present.
